# Differences in Patients’ and Surgeons’ Expectations before Shoulder Stabilization Surgery

**DOI:** 10.3390/jcm10204661

**Published:** 2021-10-11

**Authors:** Katrin Karpinski, Fabian Plachel, Christian Gerhardt, Tim Saier, Mark Tauber, Alexander Auffarth, Doruk Akgün, Philipp Moroder

**Affiliations:** 1 Centrum für Muskuloskeletale Chirurgie, Klinik für Schulter- und Ellenbogenchirurgie, Charité Universitätsmedizin Berlin, 13353 Berlin, Germany; katrin.karpinski@t-online.de (K.K.); fabian.plachel@charite.de (F.P.); doruk.akguen@charite.de (D.A.); 2 St. Vincentius-Klinik Karlsruhe, 76137 Karlsruhe, Germany; christian.gerhardt@vincentius-ka.de; 3 BG Unfallklinik Murnau, 82418 Murnau am Staffelsee, Germany; tim.saier@me.com; 4 ATOS Klinik München, 81925 München, Germany; mark.tauber@atos.de; 5 Universitätsklinikum Salzburg, A-5020 Salzburg, Austria; a.auffarth@salk.at

**Keywords:** shoulder instability, shoulder stabilization, patients’ expectations, surgeons’ expectations

## Abstract

Purpose: The primary goal of shoulder stabilization procedures is to re-establish stability and many surgeons measure the success after shoulder stabilization surgery only by the absence of re-dislocation. However, patients might also suffer from pain, loss of range of motion and strength as well as anxiety and stigmatization and therefore have other expectations from a stabilization surgery than just a stable shoulder. Purpose of this study was to analyze if surgeons know what their patients typically expect from a shoulder stabilization surgery. Furthermore, the aim was to analyze the influence of various factors on patients’ expectations. Materials and Methods: 204 patients with a diagnosis of shoulder instability scheduled for surgical treatment were included in this prospective multicentric study. Preoperatively, objective and subjective scores were obtained and patients were asked about their postoperative expectations. Additionally, 25 surgeons were interviewed with regard to what they think their patients expect from the surgery using standardized questions. Results: With regard to postoperative expectations surveyed by the Hospital for Special Surgery questionnaire (HSS), the most important goal to achieve for the patients was ‘stopping the shoulder from dislocation’, followed by ‘to improve the ability to exercise or participate in sports’ and ‘being the shoulder to be back the way it was before the issue started’. The ranking of factors for patients was ‘stability’ as the most important to achieve, followed by ‘movement’, ‘strength’, ‘pain’ and ‘cosmetics’. For surgeons, the order was ‘stability’ (*p* = 0.004 **), ‘movement’ (*p* = 0.225), ‘pain’ (*p* = 0.509), ‘strength’ (*p* = 0.007 **) and ‘cosmetics’ (*p* = 0.181). There was a significant difference between patients and surgeons with regard to gaining stability at the cost of movement (*p* = 0.001 **). Conclusion: Patients and surgeons expectations regarding outcome after surgical shoulder stabilization procedures are quite similar with limited topics of disagreement. Generally, surgeons tend to overrate the importance of stability at the costs of other factors.

## 1. Introduction

With an incidence of 11–32 shoulder dislocations per 100,000 people per year, the glenohumeral joint is the joint most susceptible to instability [1,2,3]. Shoulder instability can either be caused by structural or functional deficiencies. While functional shoulder instability can be treated conservatively [4], instability caused by structural defects often requires surgery in order to achieve stability [5]. Therapy options include repair of soft tissue defects (labral and capsular structures) as well as bony defects (glenoid bone loss or Hill-Sachs lesions), depending on the extent of the damage [6]. Various studies have shown satisfying clinical and radiological outcome after arthroscopic Bankart repair [7,8], Latarjet procedure [9,10,11] or free bone-grafting techniques [6,12,13] and patients show improved quality of life after Bankart repair [14]. The primary goal of surgical therapy options is to re-establish stability and many surgeons measure the success after shoulder stabilization surgery only by the absence of re-dislocation. However, patients might also suffer from pain, loss of range of motion and strength as well as anxiety and stigmatization and therefore have other expectations from a stabilization surgery than just a stable shoulder [15]. When expectations of surgeons and patients are not the same, it might lead to frustration on both sides, taking into account that satisfaction and objective functional outcome might differ [16,17,18].

The purpose of this study was to analyze if surgeons know what their patients typically expect from a shoulder stabilization surgery. Furthermore, the aim was to analyze the influence of various factors on patients’ expectations.

## 2. Materials and Methods

This multicentric study was conducted prospectively in four shoulder centers in two German-speaking countries. Between July 2016 and March 2018, 204 patients who were enrolled for operative instability repair of the shoulder were included in this study. Included were patients over 18 years who gave their signed informed consent. Exclusion criteria was the inability to understand the content of the patient information and consent form as well as language other than German or English. 190 patients properly completed the questionnaires. 

Before being admitted to the hospital, patients were asked to complete a questionnaire which consisted of three parts: part A included general sociodemographic data and health information, part B was comprised by pathology-specific questionnaires and part C were questions regarding own expectations of the upcoming operation.

Part A was completed by the patient independently including questions on socio-demographic aspects. Further questions included information on dexterity, relationship status, education level and health awareness. Furthermore part A included the validated Global Physical Activity Questionnaire (GPAQ) of the World Health Organization (WHO) to capture physical activity during leisure and work. The result was given in Metabolic Equivalents (METs).

Part B refered to the actual shoulder pathology and how the patient was impaired by the instability. It contained three questionnaires: the Western Ontario Shoulder Instability Index (WOSI), the ROWE score and the Subjective Shoulder Value (SSV). The WOSI score was originally published in 1998 and its primary aim was to evaluate the outcome after shoulder instability treatment [19]. The questionnaire, which was completed by the patients themselves, consisted of five parts: physical symptoms, sports/recreation/work, pain, lifestyle and emotion. A total score of 2100 can be obtained, indicating poor shoulder function. When converted, a higher percentage indicated for a good shoulder function. The ROWE score is a self-assessment questionnaire with regard to pain, motion and function (strength and stability) in patients with shoulder instability [20]. A total of 100 points could be achieved, meaning the higher the score, the better the outcome. The SSV reflected the patient’s assessment of his or her shoulder as a percentage of a healthy shoulder which would score 100% [21]. For evaluating preoperative pain a Visual Analog Scale (VAS) was used. Furthermore, patients were asked about their pathology, its cause and any previous therapy.

In part C, the patients were asked about their postoperative expectations with regard to gain of range of motion and strength, pain relief and the influence on their activities of daily life, work and sports. These questions were summarized in the Hospital for Special Surgery questionnaire (HSS) [22]. Furthermore, individual questions were asked concerning the postoperative status of the patient. Patients were asked—besides stability—how important they rated strength, pain reduction, cosmetics and motion.

They were also questioned to which extent they would accept pain, duration of immobilization, physiotherapy, skin scar length and amount as well as aftercare to obtain a stable shoulder. Besides, patients were asked how important sympathy, appearance, experience, skills and empathy were to them when choosing a surgeon. The patients were supported by clinical research assistant to fill out this questionnaire.

Twentyfive specialized shoulder surgeons from the same institutions the patients were treated at were asked to also fill out questionnaire C based on what they believe that their own patients expected from instability surgery.

### Statistical Analysis

Statistical analysis was performed using the SPSS software version 27 (IBM-SPSS, New York, NY, USA). Prior to the beginning of the study a power analysis was conducted using the program GPower 3.1 (Heinrich Heine University, Düsseldorf, Germany). For providing a power of 80% with α = 5%, a trial with 200 patients and 25 surgeons (8:1) was necessary.

The Kologormov-Smirnoff test was used to test all data for normal distribution. Descriptive statistics are presented as mean ± standard deviation (SD) if not otherwise stated. A correlation analysis between sociodemographic data and patients’ expectations was calculated using the Spearman’s correlation coefficient.

The Mann-Whitney-U test was calculated to determine whether there was a difference between the expectations of surgeons and patients. Statistical significance was indicated by a *p* value of less than 0.05 (* *p* ≤ 0.05, ** *p* ≤ 0.01).

## 3. Results

### 3.1. Patient Demographics

The mean age at time of surgery was 35.0 years ± 11.2 (range 17–70). 147 patients were male (77%), 43 female (23%). The average BMI was 24.9 ± 4.1 (range 17.5–45.9). The dominant shoulder was affected in 99 cases (52%). 83% were compulsorily insured, while 17% had private insurance. With regard to their relationship status, 47% of patients stated to be single, 28% were in a relationship, 23% were married and 2% divorced. 25% of patients declared ‘apprenticeship’ as their highest education level, 29% ‘high school graduation’, 11% ‘bachelor’, 11% ‘master’, 3% ‘doctorate’ and 21% ‘other’. Concerning their health, 25% of patients answered they would take care ‘a lot’, 55% ‘reasonably’, 19% ‘moderately’ and 1% ‘not at all’.

### 3.2. Preoperative Status

The average physical activity as calculated by the GPAQ was 197 ± 221 MET-minutes/week (range 0–1712). The average WOSI score preoperatively was 42 ± 18% (range 6–92%) and the mean ROWE score was 61 ± 20 (range 0–100). The mean SSV amounted 52 ± 22 (range 0–100). With regard to the preoperative VAS, the average score was 3.2 ± 2.5 (range 0–10). 124 of all patients (65%) sustained from at least one dislocation of their shoulder, 40 patients (21%) reported subluxations and 26 (14%) suffered from apprehension only. Over all, 42 patients (22%) had an operative treatment before. On average, patients suffered 3 ± 5 (range 0–30) years from instability before operation. 

### 3.3. Patients’ and Surgeons’ Expectations

**Figure 1:**
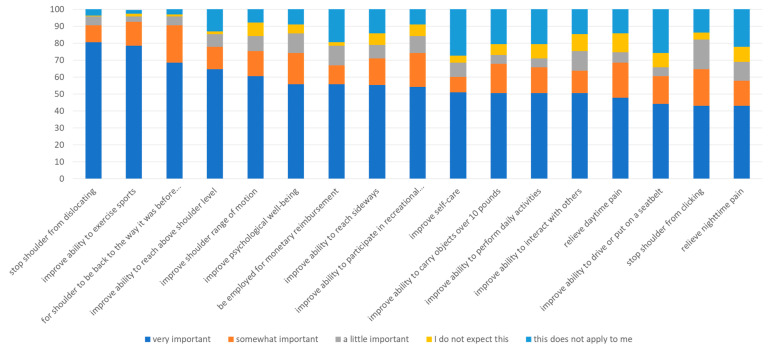
Patients’ expectations using the Hospital for Special Surgery Shoulder Surgery Expectations Survey‘ (HSS).

With regard to postoperative expectations surveyed by the HSS questionnaire [22], the most important goal to achieve for the patients was ‘stopping the shoulder from dislocation’ (very important 81%, somewhat important for 10%), followed closely by ‘to improve the ability to exercise or participate in sports’ (very important for 78%, somewhat important for 14%). To come third was ‘the shoulder to be back the way it was before the issue started’ (very important for 68%, somewhat important for 22%). The detailed evaluation of the Hospital for Special Surgery Shoulder Surgery Expectations Survey is provided within Figure 1.

Furthermore, patients were asked which of the following factors ‘strength’, ‘pain’, ‘cosmetics’, ‘movement’ and ‘stability’ was the most important for them. On the first place was ‘stability’ (64% rank 1, 20% rank 2, 8% rank 3), on the second position ‘movement’ (17% rank 1, 51% rank 2, 19% rank 3) and on the third ‘strength’ (5% rank 1, 17% rank 2, 49% rank 3). ‘Pain’ gained rank 4 and ‘cosmetics’ was on rank 5.

**Figure 2:**
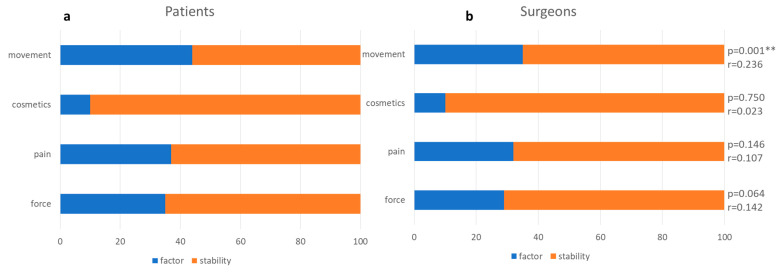
Patients’ (**a**) and surgeons’ (**b**) ratio of importance between stability and other outcome parameters. There was a significant difference in rating movement in comparison to stability between patients and surgeons (** *p* ≤ 0.01).

For surgeons, ‘stability’ was on rank 1 (96% rank 1, 4% rank 2) (*p* = 0.004 **, r = 0.200), the second place took ‘movement’ (64% rank 2, 28% rank 3, 8% rank 4) (*p* = 0.225, r = 0.085), on the third place was ‘pain’ (8% rank 1, 16% rank 2, 40% rank 3, 36% rank 4) (*p* = 0.509, r = 0.046), to come fourth was ‘strength’ (4% rank 1, 8% rank 2, 28% rank 3, 60% rank 4) (*p* = 0.007 **, r = 0.188) and fifth ‘cosmetics’ (100% rank 5) (*p* = 0.181, r = 0.094). A comparison of the importance of achieving stability at the cost of other factors for patients and surgeons is displayed in Figure 2.

**Figure 3:**
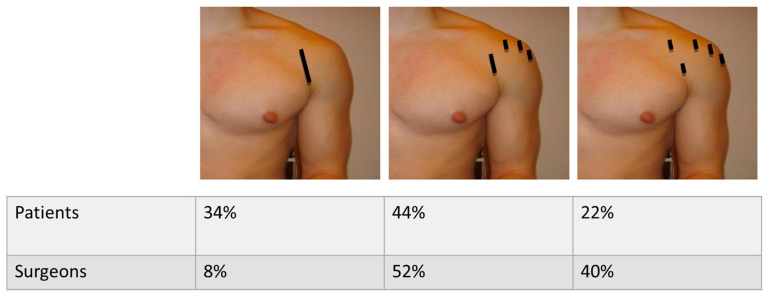
Scars (black lines) patients would prefer for instability repair and what surgeons keep in mind (*p* = 0.005, r = 0.193).

Concerning cosmetics, 34% of all patients would prefer one scar of 5 cm length, 44% of all patients would prefer three scars of 1cm length and one scar of 2 cm length and 22% of all patients would prefer five scars of 1cm length. The scars were illustrated on images for a better presentation for the patients and surgeons. There was a significant difference in what scar configuration surgeons expected being chosen by the patients (*p* = 0.005, r = 0.193) (Figure 3).

**Figure 4:**
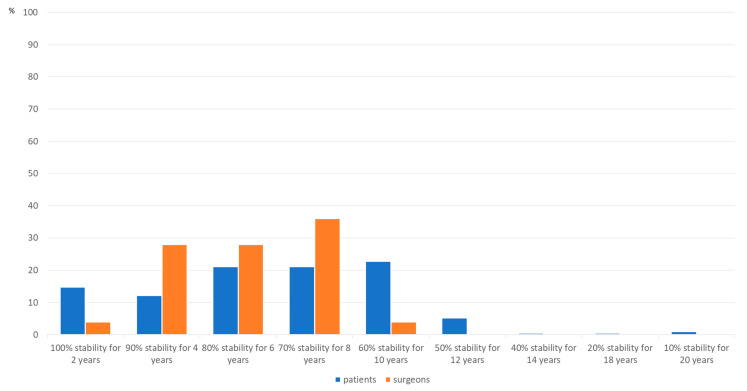
Preferred stability over time (*p* = 0.163, r = 0.095).

Figure 4 displays the ratio of importance between effective stability and duration of stability.

**Figure 5:**
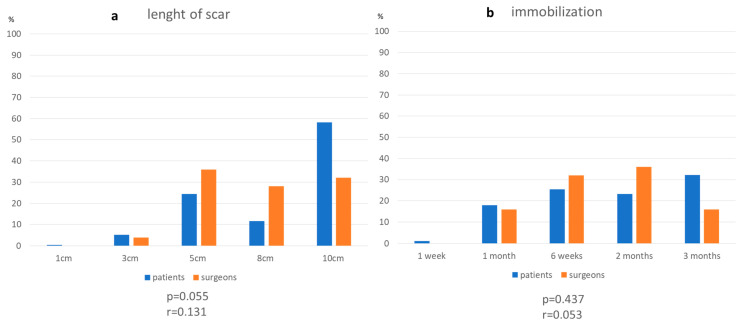

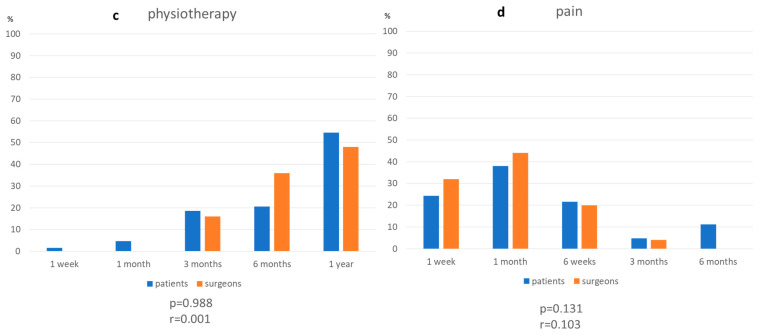

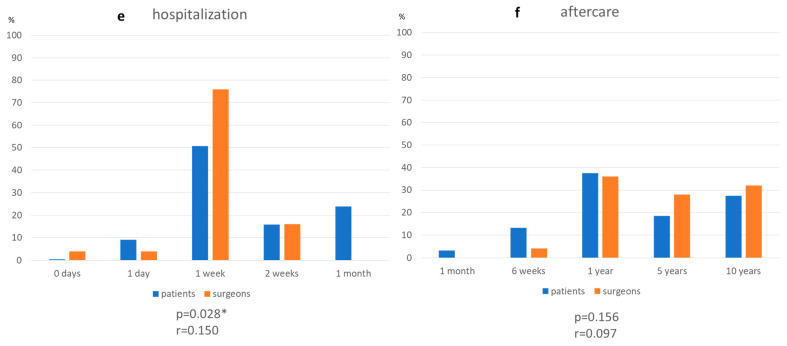
Patients’ acceptance for 100% shoulder stability and surgeons’ perspective with regard to length of scar (**a**), immobilization (**b**), physiotherapy (**c**), pain (**d**), hospitalization (**e**) and aftercare (**f**). (* *p* ≤ 0.05).

What patients would accept in terms of scars, immobilization, physiotherapy, postoperative severe pain, hospitalization and aftercare for reaching a 100% stable shoulder and surgeon’s perspective is displayed in Figure 5a–f.

**Figure 6:**
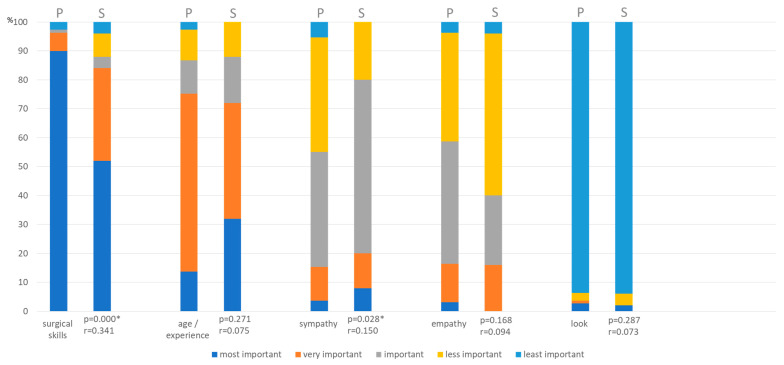
Opinion about most important factor for choosing surgeon (P = patients, S = surgeons). (* *p* ≤ 0.05).

Patients and physicians were interviewed about the most important factor when it comes to choosing the surgeon. The outcome can be seen in Figure 6.

Stopping the shoulder from dislocation was less important the older the patients were and the higher VAS pain score was preoperatively. ‘Stability’ got a higher rank the lower the BMI, GPAQ or VAS respectively the higher WOSI or ROWE score was and if patients had more dislocations (in comparison to just apprehension). Full range of motion was more important for patients with a bad WOSI or ROWE score and the shorter they had their symptoms. The importance of gaining strength correlated with a bad preoperative WOSI, ROWE, VAS score or SSV and an onset of symptoms that was longer ago. The factor ‘force’ was ranked higher the younger the patients were, if they were male and single. The ability of participating in sports was more important for younger patients. The higher the VAS score preoperatively, the older the patient was, the longer they had their symptoms and the worse preoperative scores were the more important pain relief was for these patients. Pain relief was also ranked higher if the patient was compulsory insured. Patients would accept physiotherapy the longer, the lower their SSV was and the shorter they had their symptoms. With regard to postoperative hospitalization, patients would accept a longer period of time if they had a bad SSV or VAS preoperatively and a shorter duration of onset of symptoms. Dexterity, education or how much the patient cares about health had no significant influence on the expectations or what patients would accept to obtain 100% shoulder stability. Detailed information can be obtained in Table 1 and Table 2.

## 4. Discussion

The analysis showed that subjective expectations of the patients with regard to their surgical outcome did not differ widely from the surgeons’ believe regarding the importance of regaining a stable shoulder, though surgeons tended to overrate the importance of stability at the costs of other factors.

When it comes to the most important postoperative outcome goal, patients as well as surgeons voted ‘stability’ on rank 1. Patients and surgeons also agreed on the second place ‘movement’. When it comes to rank 3, patients chose ‘strength’, while surgeons voted for ‘pain’. When it comes to rank ‘stability’ against other factors, Figure 2 shows that full range of motion was more important for the patients than surgeons think. This is also confirmed by the HSS, where patients ranked the ability to exercise or participate in sports even slightly higher than stopping the shoulder from dislocation (Figure 1). That should be taken into account, as some surgical stabilization techniques might lead to a loss of range of motion [9,11,13]. Therefore this topic should be discussed with the patient before choosing the adequate type of surgery. 

Surgeons tend to overrate the ultimate goal of stabilizating the shoulders of their patients. While gaining stability was especially important for young patients with a lower BMI, with a lower VAS preoperatively, for older patients, getting rid of pain was more important than gaining stability. In contrast, for the stereotype of a young male single patient, gaining force was more important. This underlines once more the necessity of clearly addressing the patient’s individual needs before surgery.

The worse preoperative scores such as WOSI, ROWE and SSV were and the shorter patients had their symptoms, the more important it was to relief pain and gain range of motion and force with stability remaining more in the background. This counts also for a higher VAS preoperatively. The likely explanation is that patients presenting with an acute shoulder instability have stronger functional limitations and pain which they want to get rid-off, while the focus for patients with a chronic instability is the instability itself as pain and loss of function are often limited.

It was generally agreed that cosmetics had no importance when it comes to obtaining a stable shoulder. Surgeons overestimate the importance of scar configuration for their patients (Figure 5a). Figure 3 shows no trend for a preferable scar configuration among patients, with most patients willing to accept longer scars to obtain a stable shoulder. 

With regard to the length of immobilization, physiotherapy and severe pain patients would undertake for gaining a 100% stable shoulder, surgeons rightly assessed their patients’ expectations (Figure 5b–d). 96% of all interrogated surgeons think that patients will accept at least one year of aftercare, while 84% of the patients would be willing to do so (Figure 5f). In general, patients would be willing to undertake a longer period of physiotherapy or hospitalization, the lower their preoperative SSV was, the higher their VAS was and the shorter their symptoms lasted preoperatively, possibly indicating higher psychological stress. 

When it comes to the most important factor when choosing the surgeon, ‘surgical skills’ was even higher rated and ‘sympathy’ lower than surgeons thought (Figure 6).

Plath et al. also investigated the expectations on the surgical outcome after shoulder instability repair and demonstrated that they are generally high, especially in athletes. They also highlighted that the surgeon must take into account not only the surgical procedure and known risk factors for failure but at the same time the individual expectations to improve overall satisfaction [23]. Patients’ expectations of shoulder surgery was as well investigated by Mancuso et al. [22]. By asking 409 patients, they developed a patient-derived shoulder surgery expectations survey (HSS), taking into account various goals of the patient such as pain relief, gain of range of motion or reestablishing activities of daily life as well as sports. The study shows that patients’ expectations with regard to the postoperative outcome vary by demographics, diagnosis as well as functional status. Within the instability group, the aim to return to sports (80%) was even more important for the patients than avoiding re-dislocation (53%). Also in the present study improving the ability to exercise sports was ranked very high compared to gaining stability (Figure 1). The HSS study could provide a template for surgeons to guide a discussion with their patients about realistic and unrealistic outcome goals what might improve shared decision making [22]. Especially with regard to elective surgical procedures, this could enhance the process of obtaining informed consent. Mancuso et al. could demonstrate that patients expectations agreed the more with the surgeons’ the better they were informed [24].

The pre-treatment interview plays an essential role. The more the patient was informed before surgery, the better their expectations resonated with the ones of the surgeon. A higher level of satisfaction will be the consequence [24]. Also other studies suggest that the better patients are educated about the planned procedure, the better the subjective postoperative outcome will be [25,26,27]. This underlines once more the necessity of a comprehensive and clear provision of information to the patient about their pathology, the planned surgery and expected outcome. The surgeon’s experience on the realistic outcome and the priorities of the patient should be discussed within the preoperative setting. One should always take into account that patients might not be aware of their underlying pathology and just seek for an operative solution with low self-responsibility which will inevitably lead to unrealistic expectations [28]. To avoid disappointment on both sides, all the things mentioned should be addressed within the pre-treatment interview. 

This study also has its limitations. First of all, patients tend to set their actual expectations higher, fearing that otherwise this might lead to poorer effort by the surgeon. The fact that patients might answer in favor of the surgeon should be taken into account. Besides, every surgeons’ pre-treatment interview will vary individually, depending on their time, experience and maybe also sympathy for the patient. Furthermore, the individual rankings might have been misunderstood by the surgeons, as they didn’t get help by a clinical research assistant to answer the questions.

## 5. Conclusions

Patients and surgeons expectations regarding outcome after surgical shoulder stabilization procedures are quite similar with limited topics of disagreement. Generally, surgeons tend to overrate the importance of stability at the costs of other factors. When planning a therapy, surgeons should always take into account the subjective expectation s and individual needs of their patients. The communication about a possible divergence with regard to the expectation of surgeons and patients concerning the postoperative outcome might allow an optimization of the therapy algorithm, an increase of compliance of the patients as well as the development of new therapy approaches. All these efforts might lead to a better satisfaction on both sides postoperatively.

## Figures and Tables

**Table 1:** Correlation coefficients of the HSS and patients’ preoperative data (* *p* ≤ 0.05, ** *p* ≤0.01, n.s. = no significance).

	Daytime Pain	Nighttime Pain	Range of Motion	Stability	Force	Sports	Postoperative Physiotherapy	Postoperative Hospitalization
**age**	−0.207 **	n.s.	n.s.	0.144 *	n.s.	0.212 *	n.s.	n.s.
**dominant shoulder**	n.s.	n.s.	n.s.	n.s.	n.s.	n.s.	n.s.	n.s.
**GPAQ**	n.s.	n.s.	n.s.	n.s.	n.s.	n.s.	n.s.	n.s.
**WOSI**	0.440 **	0.377 **	0.193 **	n.s.	0.330 **	n.s.	n.s.	n.s.
**ROWE**	0.274 **	0.271 **	0.247 **	n.s.	0.264 **	n.s.	n.s.	n.s.
**SSV**	0.211 **	0.206 **	n.s.	n.s.	0.156 *	n.s.	−0.145 *	−0.163 *
**VAS**	−0.388 **	−0.349 **	n.s.	0.201 **	−0.146 *	n.s.	n.s.	0.215 **
**duration of symptoms**	n.s.	0.144 *	0.208 **	n.s.	0.398 **	n.s.	−0.175 *	−0.184 *

**Table 2:** Correlation coefficient of the ranking of factors and patients’ demographics and scores (* *p* ≤ 0.05, ** *p* ≤ 0.01, n.s. = no significance).

	Force	Pain	Stability	Movement	Cosmetics
**age**	0.175 *	0.146 *	0.162 *	n.s.	n.s.
**Gender (male/female)**	0.231 **/n.s.	n.s.	n.s.	n.s.	n.s.
**BMI**	n.s.	n.s.	*p* = 0.203 **	n.s.	n.s.
**Relationship status (single/relationship)**	*p* = 0.147 */n.s.	n.s.	n.s.	n.s.	n.s.
**Insurance (compulsory/private)**	n.s.	0.155 */n.s.	n.s.	n.s.	n.s.
**GPAQ**	n.s.	n.s.	0.147 *	n.s.	n.s.
**WOSI**	n.s.	0.208 **	0.155 *	n.s.	n.s.
**ROWE**	*p* = 0.157 *	0.297 **	0.218 *	n.s.	n.s.
**SSV**	n.s.	0.189 *	n.s.	n.s.	n.s.
**VAS**	n.s.	0.292 **	0.273 **	n.s.	n.s.
